# Why Hantavirus Prevalence Does Not Always Increase With Host Density: Modeling the Role of Host Spatial Behavior and Maternal Antibodies

**DOI:** 10.3389/fcimb.2020.536660

**Published:** 2020-09-29

**Authors:** Jonas Reijniers, Katrien Tersago, Benny Borremans, Nienke Hartemink, Liina Voutilainen, Heikki Henttonen, Herwig Leirs

**Affiliations:** ^1^Evolutionary Ecology Group, Biology Department, University of Antwerp, Antwerp, Belgium; ^2^Active Perception Lab, Department of Engineering Management, University of Antwerp, Antwerp, Belgium; ^3^Agentschap Zorg en Gezondheid, Government Administration, Brussels, Belgium; ^4^Department of Ecology and Evolutionary Biology, University of California, Los Angeles, Los Angeles, CA, United States; ^5^Interuniversity Institute for Biostatistics and Statistical Bioinformatics, Hasselt University, Hasselt, Belgium; ^6^Theoretical Ecology, Institute for Biodiversity and Ecosystem Dynamics, University of Amsterdam, Amsterdam, Netherlands; ^7^Biometris, Wageningen University and Research, Wageningen, Netherlands; ^8^Department of Virology, University of Helsinki, Helsinki, Finland; ^9^Terrestrial Population Dynamics, Natural Resources Institute Finland, Helsinki, Finland

**Keywords:** hantavirus, Puumala virus, spatial behavior, maternal antibodies, bank vole

## Abstract

For wildlife diseases, one often relies on host density to predict host infection prevalence and the subsequent force of infection to humans in the case of zoonoses. Indeed, if transmission is mainly indirect, i.e., by way of the environment, the force of infection is expected to increase with host density, yet the laborious field data supporting this theoretical claim are often absent. Hantaviruses are among those zoonoses that have been studied extensively over the past decades, as they pose a significant threat to humans. In Europe, the most widespread hantavirus is the Puumala virus (PUUV), which is carried by the bank vole and causes nephropathia epidemica (NE) in humans. Extensive field campaigns have been carried out in Central Finland to shed light on this supposed relationship between bank vole density and PUUV prevalence and to identify other drivers for the infection dynamics. This resulted in the surprising observation that the relationship between bank vole density and PUUV prevalence is not purely monotonic on an annual basis, contrary to what previous models predicted: a higher vole density does not necessary result in a higher infection prevalence, nor in an increased number of humans reported having NE. Here, we advance a novel individual-based spatially-explicit model which takes into account the immunity provided by maternal antibodies and which simulates the spatial behavior of the host, both possible causes for this discrepancy that were not accounted for in previous models. We show that the reduced prevalence in peak years can be attributed to transient immunity, and that the density-dependent spatial vole behavior, i.e., the fact that home ranges are smaller in high density years, plays only a minor role. The applicability of the model is not limited to the study and prediction of PUUV (and NE) occurrence in Europe, as it could be easily adapted to model other rodent-borne diseases, either with indirect or direct transmission.

## Introduction

For wildlife diseases, to make an inference on infection prevalence and the force of infection to reservoir and spillover hosts (e.g., humans), host population density is often used as a proxy (e.g., Davis et al., 2005). When the rate at which an infectious disease is transmitted depends on the density of the host population, theory predicts prevalence in a closed, stable population to increase with population density (Anderson and May, 1979; Keeling and Rohani, 2008). While this holds for most human infections, the effect of density on infection prevalence and force of infection is more complex for wildlife diseases. Because wildlife populations are generally unstable, smaller and localized, they tend to be influenced by stochasticity more. Also, animal hosts often have a short generation time, and because reproduction and survival is strongly linked to environmental factors, the composition and density of populations tend to fluctuate at different temporal scales (Hudson et al., 2002; Davis et al., 2005; Lloyd-Smith et al., 2005; Alexander et al., 2012; Allen et al., 2012). Behavioral changes, for example due to changes in reproductive status or density, can have a direct effect on transmission through changes in the number and type of contacts (Ezenwa, 2004; Nunn et al., 2011; Borremans et al., 2016).

Because of this complexity, it can be difficult to understand the relationship between host density, infection prevalence and force of infection to humans. Moreover, in wildlife disease systems there is often a lack of longitudinal infection data with sufficient (temporal) resolution, as this requires long-term labor-intensive sampling of a large number of host animals in the field (Allen et al., 2012).

Hantaviruses are among the best studied (re-)emerging zoonotic pathogens, whose transmission is considered to be density-dependent (Mills et al., 1999; Madhav et al., 2007). For several hantaviruses, however, it was found that host density and prevalence are not always positively correlated on a short temporal scale (Niklasson et al., 1995; Escutenaire et al., 2000; Davis et al., 2005; Olsson et al., 2005; Linard et al., 2007; Tersago et al., 2009; Kallio et al., 2010; Luis et al., 2015). Age-dependent infection risk, combined with seasonal variations in age structure, has long been assumed to be the reason for this: seasonal breeding results in periodic increases of susceptible individuals that temporarily decrease prevalence while simultaneously increasing the transmission rate, resulting in a delayed density-dependent effect on prevalence (Mills et al., 1999; Mills, 2005).

While the relationship between prevalence and density may be complex on a short timescale (within the year) (Adler et al., 2008), density-effects on prevalence should be more apparent on longer, multi-annual timescales. Indeed, a key pattern that has been observed is that when transmission rates increase with host density, the total number of infection events during and after a high-density period (long-term) is larger, even when direct (short-term) density-dependence of prevalence is not observed (Mills et al., 1999). This seems to hold for several new and old world hantaviruses occurring in fluctuating rodent species (for reviews see Mills et al., 2010; Olsson et al., 2010; Heyman et al., 2012), and following this observation, it has been possible to associate the main drivers of the rodent host density with hantavirus disease epidemics in humans. This was done for Puumala Orthohantavirus (PUUV) in temperate Europe (Clement et al., 2009; Schwarz et al., 2009; Tersago et al., 2009, 2011). In Finland, however, annual patterns of increasing vole density do not seem to translate unequivocally to increased PUUV prevalence (Kallio et al., 2010) (see also Supplementary Figure 1).

**Figure 1:**
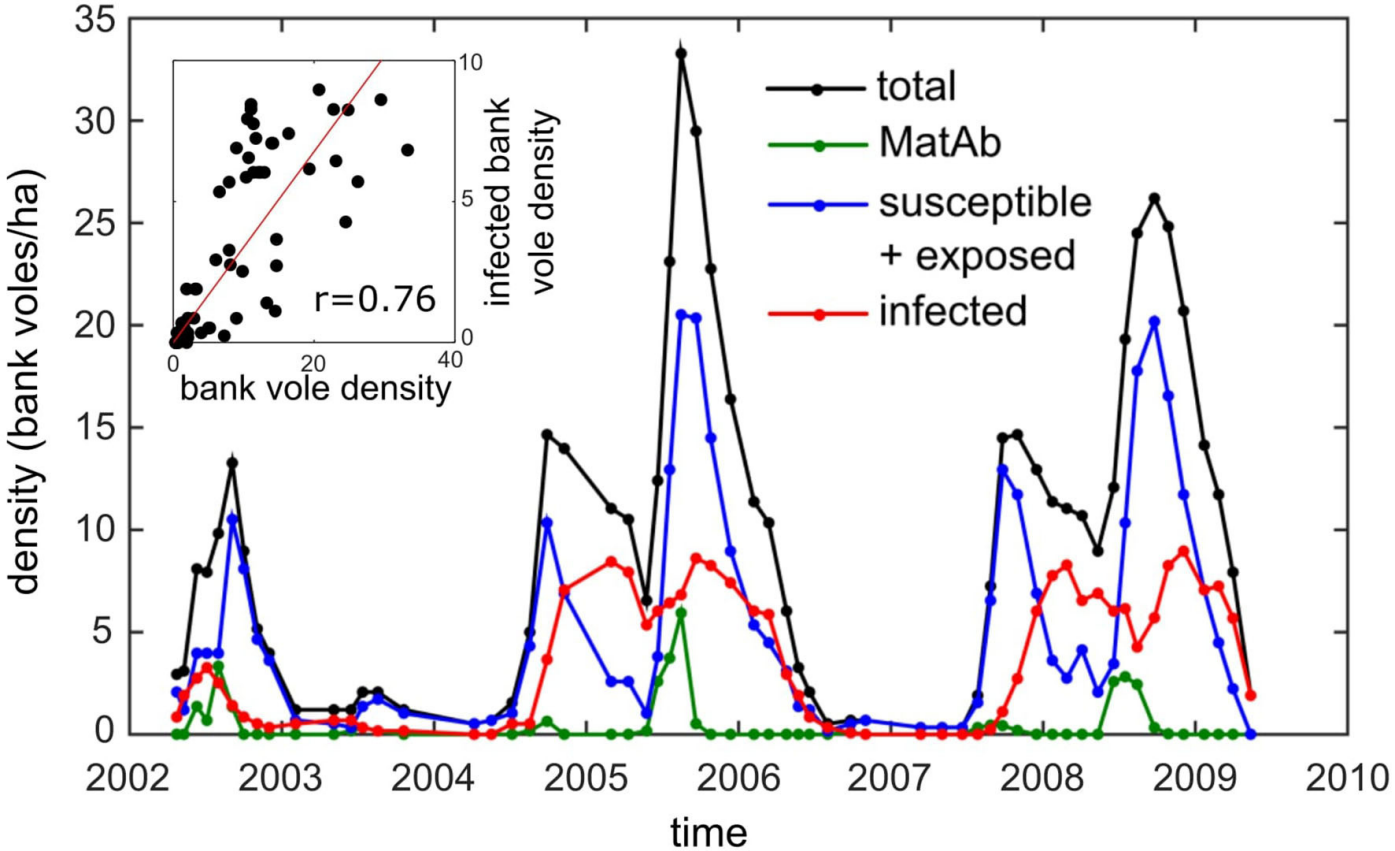
The density of bank voles (black) and the density of the different infection status categories: susceptible (and exposed) for PUUV infection (blue), infectious (red), and carrying maternal antibodies (green) on the core 5.8 ha study grid (data from Voutilainen et al., 2016). Inset: the correlation of the overall and infected bank vole densities (*r* = 0.76, *p* < 0.0001).

PUUV is a hantavirus carried by bank voles (*Myodes glareolus*) throughout large areas of the European mainland. In humans, PUUV causes nephropathia epidemica (NE), which is a mild form of hemorrhagic fever with renal syndrome (HFRS) (Brummer-Korvenkontio et al., 1980; Vaheri et al., 2013). Humans get infected through contact with infected bank voles or their excreta. PUUV can survive in the environment for long periods after shedding, and for this reason, indirect transmission is assumed the most important infection route, to other voles as well as to humans (Kallio et al., 2009). Within its distribution range, bank vole densities fluctuate among years, and these variations may be irregular or periodic, depending on the location and the top-down or bottom-up processes driving them. In temperate Europe, the fluctuations in bank vole abundance are irregular and mainly driven by food availability in the preceding year, while in the boreal zone (e.g., Finland) vole density fluctuates in a regular, cyclic fashion, thought to be driven by predator-prey interactions (Hanski et al., 2001; Korpimäki et al., 2005; Korpela et al., 2014). Because NE causes a heavy disease burden in Finland (Vaheri et al., 2013), the most extensive longitudinal dataset on PUUV has been collected here. There are increasing and declining trends in the cyclicity in Finland (Korpela et al., 2013), and our study period coincided with the period of strong cyclicity and record numbers of NE cases in Finland. The dataset of Central Finland shows that the relationship between density and prevalence is non-monotonic in this region (Figure 1): in the years with highest vole density (peak years), the abundance of infectious bank voles does not peak but attains about the same level as in the preceding increase year.

This observation is supported by another independent record: the number of human NE cases reported during the same time period. Although the number of cases can be expected to be proportional to bank vole density, it is not higher in a bank vole peak year but often even slightly lower than in increase years (Kallio et al., 2009). Even though an overall positive relationship between bank vole density and NE cases was found over a 14-years period, these data showed that increased density does not necessarily result in a higher NE incidence.

In order to explain this discrepancy, Kallio et al. (2009, 2010) put forward possible explanations. They pointed at transient immunity through maternal antibodies (MatAb) as a potential cause. Infected females transfer MatAb to their offspring, which are then temporarily protected against PUUV (Kallio et al., 2006b). In a peak year following an increase year, the seroprevalence in overwintered voles in spring approaches 100% (see Supplementary Material), and consequently most of the young born to these females should have maternal antibodies. This influx of maternally protected newborns may delay transmission beyond the abundance peak, hence reducing the infection prevalence (Garnier et al., 2014). The existence of MatAb immunity in juveniles has been described in multiple hantavirus studies in both US and Europe (for an overview see Kallio et al., 2010).

Another explanation may be that the spatial behavior of bank voles varies with density. In Finland, it has been observed that in peak years, when the abundance is high already at the start of the breeding season, breeding female voles have smaller territories compared to low-abundance years (Koskela et al., 1999; Eccard et al., 2011a), probably to limit the number of (hostile) contacts. Such decrease in territory/home range size may affect contacts among voles, impede infection transmission and may, if not in combination with the influx of immune newborns, be responsible for a reduced prevalence at higher abundances. The presence of acute and chronic infections in bank voles was also mentioned by Kallio et al. (2009) as a possible contributing factor. It is commonly assumed that viral shedding during the acute infection period is higher compared to the chronic stage of infection (Gavrilovskaya et al., 1990; Sauvage et al., 2003; Hardestam et al., 2008), though recently Voutilainen et al. (2015) showed that, at least for some transmission routes (urine and feces), the amount of virus shed possibly does not decrease over time.

In this paper we tested two hypotheses as possible causes for the reduced density of infectious voles (and the reduced number of human NE-cases) in a peak year: the transient immunity of maternal antibodies and the spatial behavior of bank voles during a peak year. To this end, we advanced a new spatially-explicit model for PUUV dynamics that includes maternally derived waning immunity and reduced home ranges in high density years. We also considered different shedding patterns (constant/decreasing) and different transmission routes (direct/indirect) in the simulations, since these system parameters are difficult to assess and not well-agreed upon. This allowed us to test to what extent the conclusions drawn are sensitive to these parameters. Understanding the relationship between density and prevalence is not only of practical use as it would help to predict disease outbreaks and the possible infection risk for humans, but, as we will see further, this also allows us to deduce important model parameters, hence giving insight into the virus-host interactions driving the infection dynamics.

## Methods

### Model

Earlier hantavirus models have dealt mainly with hantaviruses in the New world, e.g., the Sin Nombre virus carried by deer mice, as they pose a significant threat to humans. A basic population dynamics model was introduced by Abramson and Kenkre (2002), who studied the spatio-temporal patterns of the infection, setting the stage for a series of models with increasing complexity (Aguirre et al., 2002; Abramson and Kenkre, 2003; Giuggioli et al., 2005; Kenkre, 2005). This resulted in the so-called liquid-solid model (Kenkre et al., 2007), which considers two extreme types of movement that the juveniles and adults perform in the field (freely diffusing and static); the home range itself does not vary. These models allow for the environmental conditions to change both in time and space, by means of the so-called carrying capacity, which characterizes the host densities that the system can sustain.

For models of the Old world PUUV (Sauvage et al., 2003, 2007; Allen et al., 2006; Wesley et al., 2010), the focus was rather on the temporal than on the spatial dynamics of the PUUV spread. As bank vole population density varies greatly within and between years, these models intend to describe the temporal dynamics of vole populations and how the population dynamics affect the PUUV prevalence. The mean field approximation was assumed, which means that the transmission was not modeled as a local (spatial) process. Wolf et al. (2006) extended the model to study the effects of spatial heterogeneity on a larger scale by introducing additional compartments corresponding with (a few) other localities, thus extending their set of coupled differential equations to be calculated.

In this paper, we developed a novel spatially-explicit individual-based model and since we hypothesize that the varying spatial behavior of the bank vole is important for the virus transmission dynamics, we put great effort into modeling the bank vole movements, which depend on sex, maturation stage and the season. Therefore, each of the bank voles has to be simulated individually and the home range of each individual needs to be able to change over time and space. Because of the fact that the virus transmission is likely to be indirect (via feces, urine, saliva, Kallio et al., 2006a; Voutilainen et al., 2015, 2016), it is necessary to simulate the local build-up and the subsequent decay of virus in the environment. Model parameters on the bank vole life cycle, movement and infection dynamics were taken from the literature to fit the mid-boreal zone in Central-Finland.

Bank vole movements are modeled on an *L* × *L* square grid; each cell corresponding to a small, homogeneous landscape patch that can accumulate virus. Note that the model is essentially different from cellular automata models, since the individual bank vole is the unit of simulation and not the grid cell. The use of a grid is only a way to simulate the indirect virus transmission from an infectious bank vole to the environment to another bank vole. For each bank vole, all stochastic processes (birth, death, transmission, etc.) are modeled using a Poisson process with their respective rates. In the simulation we used a fixed time step, which was chosen small enough (Δ*t* = 1day) to capture the stochastic processes at work.

### Life Cycle

We consider four maturation stages: juveniles, subadults, dispersing adults and settled adults, of which only the settled adults can reproduce (Bondrup-Nielsen and Karlsson, 1985). We also distinguish between female and male bank voles. In the following we give a short description of the biology of the bank vole and the associated model assumptions.

#### Birth

**Figure 2:**
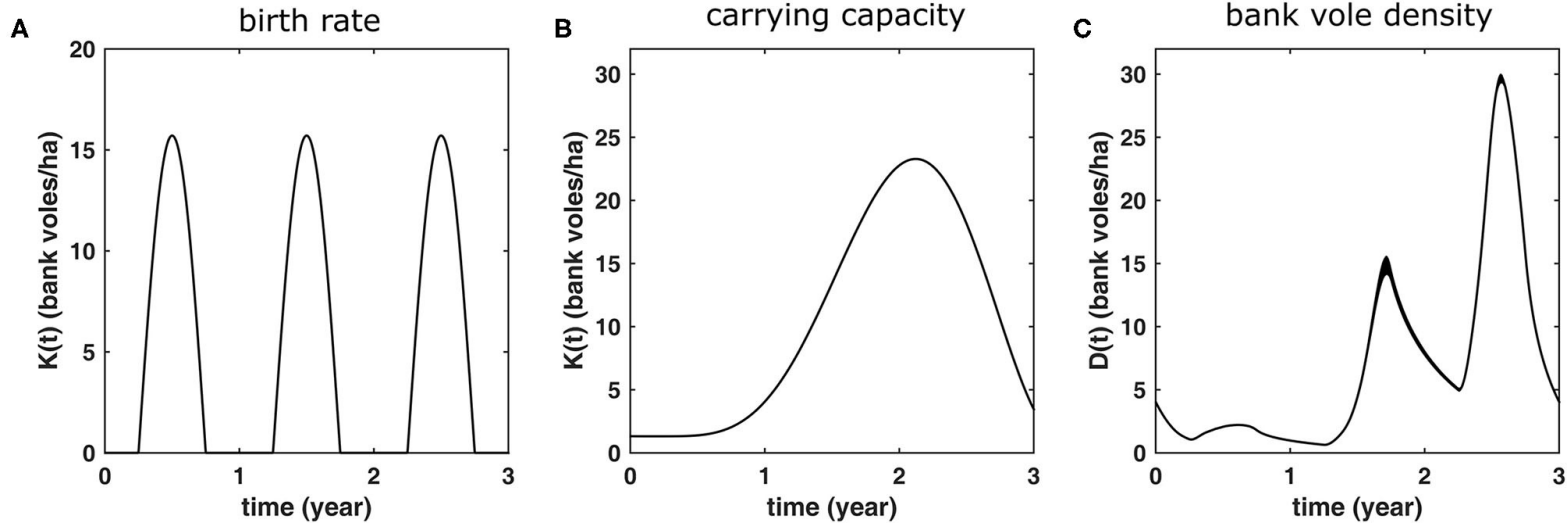
Bank vole demography as it is implemented as a 3-years cycle in the model simulations. These same conditions shown in are repeated every 3 years. **(A)** The rate at which adult females produce five-offspring litters, **(B)** the carrying capacity *K*(*t*) which is used in order to produce **(C)** population dynamics with bank vole density *D*(*t*) similar to those measured in the field (see Figure 1). The latter curve shows the average of 100 3-years cycles, with the width of the curve equals twice the standard deviation. Hence, the more variation (due to the stochastic nature of the model), the thicker the curve.

In the model, female settled adult bank voles have a time-dependent probability to produce a five offspring litter given by

(1) $$\begin{array}{l} b\left(t\right)=5\pi \text{ max}\left(\text{sin}\left[2\pi \left(t-0.25\right)\right],0\right), \end{array}$$

see Figure 2A. This birth function assumes a 6 months breeding season, from April until the end of September and implies an maximum of five litters of five offspring every season for an adult female (if it survives the whole season) (Innes and Millar, 1994). The offspring is randomly assigned a sex.

#### Maturation

After birth, juveniles stay close to their maternal site for 1 month, after which they are ready for maturation (Viitala et al., 1994). If the density of adults in the surroundings of the juvenile is not too high (adult density is rarely higher than 10 voles/ha), Ims (1987) the vole matures and becomes a dispersing adult, roaming around in search of a new place to settle. If adult density is too high, it may be beneficial for the juvenile to turn into a subadult and to postpone its maturation until the next spring, when the adult density is generally low (forced delay) (Bujalska, 1983; Bondrup-Nielsen and Ims, 1986; Prévot-Julliard et al., 1999). This way the vole does not have to fight for space with other adults, and moreover, having postponed maturation, they have a higher chance of surviving winter (Kallio et al., 2007). In the model, a juvenile can only turn into a new, dispersing adult, if the adult density *A*(*t*) in it surroundings is below 10 adult voles/ha. Subadults who postponed maturation turn into dispersing adults at the start of the next spring.

Dispersing bank voles roam around for about 2 weeks, before settling in a new place. The transition from dispersing adult to settled adult is governed by the settling rate μ = 26/year, which is the reciprocal duration of the dispersing stage. After settling, adults start reproducing (Bujalska, 1983; Bondrup-Nielsen and Ims, 1986; Norrdahl and Korpimäki, 2002).

#### Mortality

In Central-Finland, bank voles live for about 7.5 months in the wild, and hence the adult mortality rate was set to *m* = 1.6/year. For simplicity, we assumed that all voles have similar mortality rates, irrespective of their sex, infection status, or maturation stage. Mortality also depends on the carrying capacity of the environment, *K*(*t*) which in our case is the maximal bank vole density the environment can sustain. We model this carrying capacity induced mortality in a similar way to Sauvage et al. (2003), i.e., m^*^(*t*) = *m* + (*b* − *m*) * *D*(*t*)/*K*(*t*), which, in the case of constant carrying capacity *K*, would result in a bank vole density *D*(*t*) = *K*.

**Figure 3:**
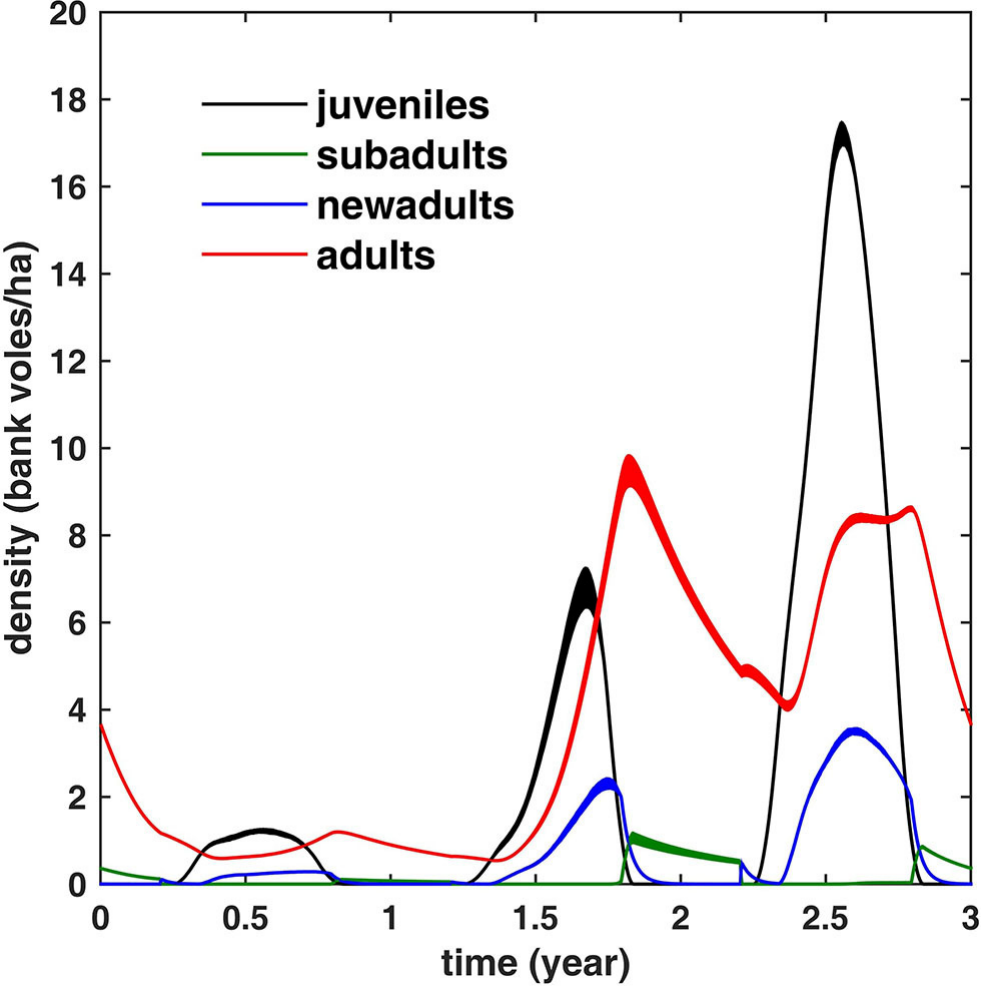
The simulated bank vole density during a 3 years cycle for different life stages (averaged over 100 cycles; the width equals twice the standard deviation). Note that adult density is limited to ≈10 bank voles/ha. In winter, all juveniles have turned either to adults or subadults; the latter mature (and turn into new adults) in spring.

We assumed the population density fluctuates with a 3 years cycle, as is typically the case for Central-Finland (see Figure 1). We modeled this by implementing a carrying capacity *K*(*t*) that varies between the years. Although the population size will never attain its equilibrium value, we use the same approach as described above and implement the mortality as m^*^(*t*) = *m* + (4.16 − *m*) * *D*^2^(*t*)/*K*^2^(*t*), where *K*(*t*) is now the time-dependent carrying capacity and 4.16 was calculated to be the effective birth rate averaged over all animals, when considering the full 3 years cycle. We considered the following functional form for *K*(*t*)

(2) $$\begin{array}{l} K\left(t\right)=c_{1}\sin^{2}\left(\frac{1}{3}\pi \left(t-c_{2}\right)\right){\text{rem}}^{2}\left(t-c_{2},3\right)+c_{3}, \end{array}$$

and have chosen *c*_1_ = 655, *c*_2_ = 0.185, and *c*_3_ = 1.3 such that the resulting simulated population dynamics curve, shown in Figure 2C, has similar maxima in an increase and a peak abundance year (14.6 and 29.6 bank voles/ha, respectively) as the mean values calculated from the field data (14.7 and 29.2, respectively) (see Figure 1). The corresponding carrying capacity *K*(*t*) is shown in Figure 2B. The densities of the voles at different life stages are shown in Figure 3.

#### Including Spatial Heterogeneities

The bank vole density and adult density vary at a local scale within the larger grid. Therefore, every bank vole “experiences” its proper bank vole (adult) density, which we calculated within a circular area with radius *R*= 200 m around the vole.

If we define respectively the distribution of bank voles on the grid **N**(*x, y*) and the circular kernel **C**(*x, y*),

$$\begin{array}{l} \text{N}(x,y)=\sum_{j}\iint \delta (x-x_{j})\delta (y-y_{j})dxdy,\\ C(x,y)=H(x^{2}+y^{2}-R^{2}), \end{array}$$

where *j* runs over all voles, and with *H*(.) the Heaviside step function, then the local adult density is obtained through convolution of both matrices, divided by the circular area π*R*^2^:

(3) $$\begin{array}{l} \text{D}\left(x,y\right)=\frac{1}{\pi R^{2}}\left(\text{N}⊗\text{C}\right)\left(x,y\right). \end{array}$$

Note that this also allows for the carrying capacity (and consequently the mortality) to be spatially-dependent. Indeed, if the environment is heterogeneous, e.g., the vegetation varies throughout the patch, this will be reflected in the local carrying capacity, which will change across the grid. This is beyond the scope of this paper, though, and we have assumed the carrying capacity to be constant over the grid.

### Movement

Bank voles have a home burrow and their movements are limited to their home range. During winter, all bank voles display more or less the same spatial behavior: they all have rather small home ranges of ~0.2 ha (Eccard et al., 2011b). At the start of the breeding season, the spatial behavior changes and the vole's home range now depends on its sex and maturation status: mature males have the largest home ranges (0.5–2 ha), which overlap with the smaller home ranges of several breeding females (0.2–0.5 ha) (Bujalska, 1983; Bondrup-Nielsen and Karlsson, 1985; Koskela et al., 1997; Eccard and Ylönen, 2007; Eccard et al., 2011a,b). During the breeding season, female adults are territorial and only tolerate their non-breeding juvenile and subadult offspring, so their home ranges are exclusive. Home ranges are thought to be negatively density-dependent, i.e., smaller in high density (peak) years (Eccard et al., 2011a); the size of the home range is set at the start of the breeding season and remains more or less fixed during the whole breeding season. At a certain point during the breeding season, when conditions are favorable, the juveniles (or overwintered subadults at the start of spring) mature and start dispersing in search of a place to settle. During this period they can travel large distances, i.e., in the order of kilometers (Bujalska, 1983), although they prefer to settle as close as possible to their native range (Viitala et al., 1994).

#### Movement Within the Home Range

Adults and juveniles move around diffusively, but are bound to their territory at position *r*_*A*_. The probability density function (pdf) of this adult (or juvenile) can then be written as the stationary solution of the harmonic model,

(4) $$\begin{array}{l} P_{A}\left(r\right)=\frac{1}{2\pi \sigma }\exp\left[-\frac{{\left(r-r_{A}\right)}^{2}}{2\sigma^{2}}\right], \end{array}$$

which is essentially a Gaussian pdf, as derived by Abramson et al. (2006). The probability is largest at the center of the territory and then decreases with increasing distance from its center. Note that for simplicity we assumed an isotropic pdf. The standard deviation σ is then related to the home range of these adult bank voles (Abramson et al., 2006; Shchipanov and Lyapina, 2008): the larger σ, the further away the vole moves and hence the larger the home range. In order to make sure that the home range of each individual (and thus σ) can change during the course of a year, we distinguish between short- and long-term movement.

**Figure 4:**
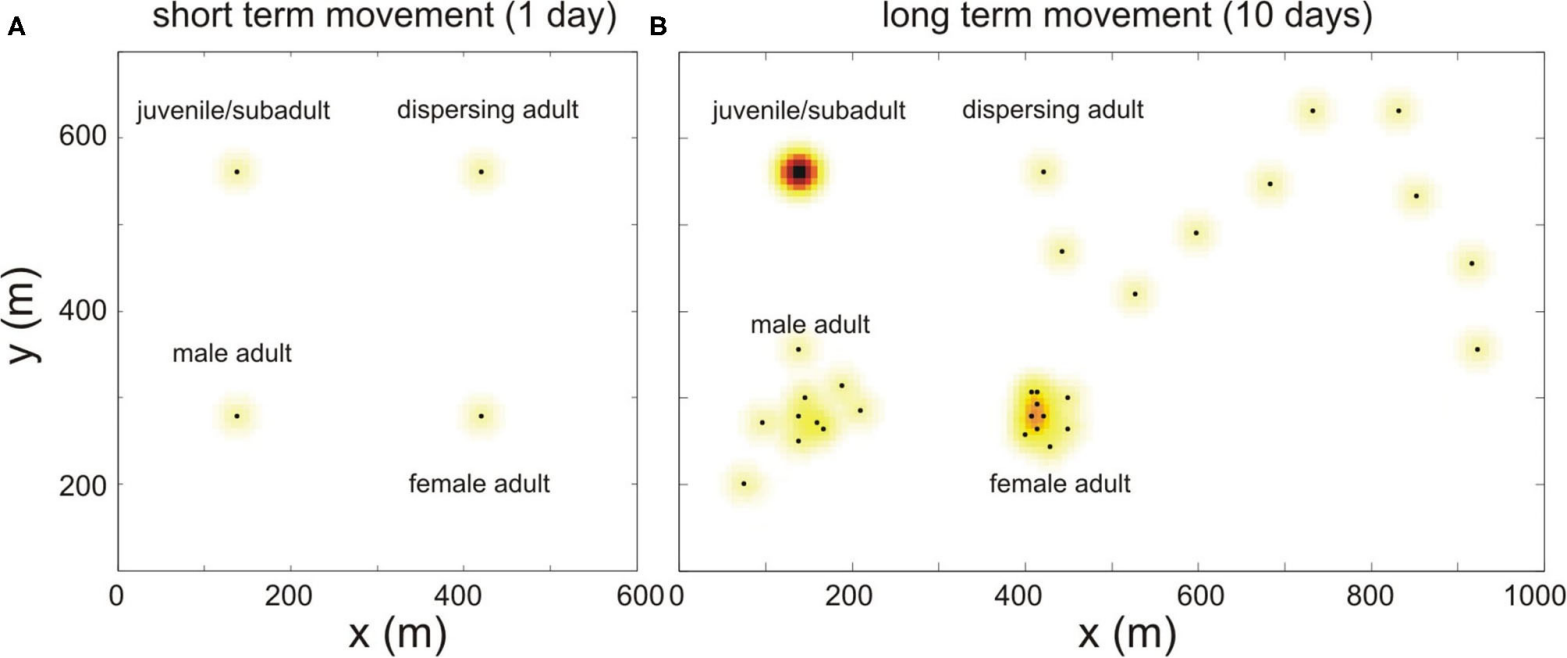
Schematic illustration of how bank vole movement is implemented in the model. **(A)** The short term movement (during 1 day) is simulated for every maturation status with the same Gaussian pdf, shown in color; the black dots represent the center of their home ranges. **(B)** The simulated movement of these same voles, during a longer period (10 days). This long term movement is modeled differently depending on the bank vole's maturation status, see text.

Short-term movement is the movement during a time step and is modeled by a Gaussian distribution with standard deviation σ_Δ*t*_ (see Figure 4A). The shape of the short term pdf is identical for all individuals and does not change over time, and it is used to determine the contact rate between animals during the time step Δ*t*.

In order to model the spatial behavior on the longer term and to arrive at a long term pdf with standard deviation σ, we simulate the spatial behavior as follows: for every time step Δ*t*, we randomly select a grid point according to a Gaussian distribution, also centered around its burrow, with variance $\sigma_{t}^{2}=\sigma^{2}-\sigma_{\Delta t}^{2}$ (see Figure 4B). By choosing this variance, the sum over the distribution of short term pdf's of each of the selected grid points over time converges to the Gaussian distribution with standard deviation σ for increasing *t*, which is the desired long term home range.

Apart from the fact that this approach gives the flexibility necessary to vary the home range size (depending on the season, sex, maturation status), it simplifies (and speeds up) the simulations significantly, while capturing the essential spatial dynamics. Moreover, it also makes sense in a biological way. Indeed, for large home ranges, the bank vole cannot travel its entire home range during a single time step, as its movement is spatially limited due to the short time interval and its limited mobility. Therefore, as it can not sample its total home range, the long-term movement has to be modeled stochastically.

#### Relating σ to Home Ranges in the Field

Using the common definition of home range as the area in which the vole spends 95% of its time, we can then easily deduce σ which corresponds to this area from Equation (4):

(5) $$\begin{array}{l} \sigma ={\left[-2\pi \text{ ln}\left(0.05\right)\right]}^{-0.5}\sqrt{\text{area}}, \end{array}$$

such that a home range (area) of 0.2 ha corresponds to σ ≈ 10.3 m.

During winter we assume that the home range size is equal for all voles, irrespective of their sex and maturation status (σ = 10 m). At the start of the breeding season, the home range size increases for adults, and the home range is different for females (0.5 ha, σ = 16 m) and males (2 ha, σ = 32 m). At the end of the breeding season, the home range of the remaining adults again shrink to the smaller home range sizes (0.2 ha, σ = 10 m). For simplicity, we chose the variance of the short term kernel equal to the variance corresponding to the smallest home ranges, i.e., σ_Δ*t*_ = 10 m. Hence, we only need to model the long term movements in the case of larger home ranges.

#### Dispersal

The above simulation applies to all settled voles, adults and juveniles, whose home range is fixed. But after maturation, new adults disperse and start moving around in search for a place to settle. We model this non-localized emigration as a random walk with turning angle distribution: at each discrete time step, the dispersing new adult travels a straight fixed distance Δ*x*, changing its angle between successive steps with Δθ with a probability

(6) $$\begin{array}{l} P\left(\Delta \theta \right)=\cos\left(\Delta \theta \right)\text{ with}-\pi <\Delta \theta <\pi , \end{array}$$

such that the vole has a higher probability to keep on moving in the same direction. We know the voles travel about 1 km during their 2 weeks period of dispersal (Viitala et al., 1994), which requires a stepsize of Δ*x* = 125 m in the case of Δ*t* = 1 day. In order to model the interaction with the environment, for simplicity, the pdf at each time step is again modeled as the short term pdf (see Figure 4B).

In our model, these new adults roam around for on average 2 weeks and those that have the least home range overlap with other adults mature first.

### Infection

#### Infectiousness

The infectiousness of a vole (the amount of virus shed per time step) over time is modeled by the infection curve *F*(*t*). Based on experiments by Hardestam et al. (2008) (laboratory conditions), who studied the amount of virus in feces, saliva and urine, we infer that after being infected, it takes about 1 week for the bank vole to become infectious; in the meanwhile, it is in the exposed state. After this week, its infectiousness increases, to reach a maximum at 25 days, after which it decreases. The vole remains chronically infectious. Hence, we model the infection curve *F*(*t*) as

$$\begin{array}{lc} F\left(t\right)=0 & t<7\text{days}\\ \;=1 & 7\text{days}<t<1\text{month},\\ \;=0.1 & \text{1 month}<t, \end{array}$$

i.e., we assume that voles undergo an acute phase of about a month, followed by a chronic infectious stage with lower shedding (10%) which lasts for the rest of their life.

In recent years, an extensive (capture-mark-recapture) dataset was collected to assess the virus shedding pattern of voles in the field (Voutilainen et al., 2015). Here too, it was shown that the virus concentration in blood decreases over time and the concentration of virus in saliva exhibited a similar acute/chronic pattern as measured in the lab (see Supplementary Material). For the virus concentration in urine and feces, the pattern was less clear and no significant decrease over time could be demonstrated. For this reason, because of uncertainty about the actual shedding pattern, we also included simulations assuming constant virus shedding, i.e., where the amount of virus shed does not decrease over time but remains constant, i.e.,

$$\begin{array}{ll} F\left(t\right)=0 & t<7\text{days}\\ \;=1 & t>7\text{days} \end{array}$$

to test the sensitivity of the simulation results to the actual shedding pattern.

Because at a very young age, juveniles spend most of their time inside the burrow, under protection of a territorial mother, their virus contamination in the environment is likely to be very limited, hence we assume that infected juveniles only start contaminating the environment at the age of 2 weeks.

#### Maternal Antibodies

A newborn is born free of infection, as there is no vertical transmission of PUUV. But if the mother is infected, the newborn will be temporarily protected by MatAb (Gavrilovskaya et al., 1990). An infected mother transfers antibodies transplacentally and via breast milk to her offspring. These maternal antibodies provide the newborns with a temporary passive immunity against infection (Gavrilovskaya et al., 1990; Dohmae et al., 1993; Bernshtein et al., 1999).

In a longitudinal study in which offspring from infected mothers were exposed to virus infected bedding at different ages, Kallio et al. (2006b) found that none of these MatAb protected voles got infected before day 80. It was only at day 105 (the next sampling point) that some of the voles showed a reappearance of Ab, which can only be attributed to an immune response to infection. If we assume that the time to seroconversion is about 3 weeks, then MatAb- protection lasts for about 80 days. We have assumed a fixed immunity period of 80 days.

#### Virus Transmission From the Infectious Vole to the Environment

The virus is transmitted through infected saliva, feces and urine. An animal can get infected through a direct (hostile or friendly) encounter with an infected animal or through contact with infected ground. Since bank voles are non-territorial during the main transmission season (winter), when transmission via fighting related wounds is improbable (Voutilainen et al., 2016), it is very likely that the indirect route is most important for virus transmission. This is also the route through which humans get infected. The virus has a non-zero survival time and hence the virus accumulates in the environment. To take this into account, we keep track of the virus accumulation on a grid: viral load at time *t* is calculated in each of the grid points (*x, y*) and is stored in the grid matrix **G**(*x, y*; *t*), which is given by

(7) $$\begin{array}{l} \text{G}\left(x,y;t\right)=\text{G}\left(x,y;t-\Delta t\right)\left(1-\xi \Delta t\right)+\beta_{1}\Delta t\underset{j}{\sum }\text{K}\left(x-x_{j},y-y_{j}\right)F_{j}\left(t\right). \end{array}$$

The first term accounts for the viral memory of the contaminated ground, whose viral load decays at a rate of ξ. The second term describes the accumulation of virus during the current time step, due to summed virus shedding of voles *j*. During this time step, these voles *j* with home range center at (*x*_*j*_, *y*_*j*_) visit the grid point (*x, y*) with probability proportional to **K**(*x* − *x*_*j*_, *y* − *y*_*j*_), which is the value of the short term pdf of vole *j* at (*x, y*). Because of the choice of the fixed short term pdf, a single kernel **K** can be used for all animals and this kernel does not change over time. The summation runs over all infectious voles so that the contribution of each vole *j* is taken into account: each vole sheds an amount of virus proportional to their infectiousness *F*_*j*_(*t*) at time *t*, resulting in a single unknown factor β_1_ in the equation (fitting method described below).

In the model, virus survival in the environment is governed by decay rate ξ. Kallio et al. (2006a) showed wild-type PUUV to maintain its infectivity at room temperature (≈22°C) for 12–15 days, so we have chosen a longevity of 2 weeks. But the longevity of PUUV was shown to depend strongly on temperature and moisture. During the winter in Northern Europe, voles spend most of their time under the snow cover in rather stable conditions (stable temperature and humidity, protected from UV light), and to account for this, we double the longevity of the virus during the winter months (from half October until half March).

#### Virus Transmission From the Environment to the Susceptible Vole

Susceptible voles move within the grid of contaminated ground **G** and can get infected. In order to calculate the probability that a susceptible vole *i*, with home range center located at grid point (*x*_*i*_, *y*_*i*_), will be infected during a single time step, one has to calculate to what extent the vole gets in contact with virus contaminated ground. Again making use of the short-term pdf describing the bank vole movement during a single time step, this can be calculated as

(8) $$\begin{array}{l} {\text{P}}_{S\to I}\left(x_{i},y_{i};t\right)=\beta_{2}\Delta t\int \int \text{G}\left(x,y;t\right)\text{K}\left(x-x_{i},y-y_{i}\right)dxdy, \end{array}$$

where β_2_ is again an unknown factor, describing the transmission efficiency from infected ground to vole (fitting method described below). The kernel **K**(*x* − *x*_*i*_, *y* − *y*_*i*_) describes to what extent the susceptible vole *i* visits each of the grid points, and **G**(*x, y*; *t*) describes the virus contamination in each of the grid points. Integration of the product of both factors over the total grid is then proportional to the amount of virus the vole came in contact with, which in turn, so we assume, is proportional to the probability that a vole becomes infected.

#### Efficient Implementation

In order to simplify and speed up calculations, we define the infectiousness matrix as

(9) $$\begin{array}{l} \text{I}\left(x,y\right)=\underset{j}{\sum }\int \int \delta \left(x-x_{j}\right)\delta \left(y-y_{j}\right)F_{j}\left(t\right)dxdy, \end{array}$$

which is essentially the distribution of the infectious voles *j* over the grid, multiplied by their infectiousness *F*_*j*_(*t*). Equation (7) can then be rewritten to

(10) $$\begin{array}{l} \text{G}\left(x,y;t\right)=\text{G}\left(x,y;t-\Delta t\right)\left(1-\xi \Delta t\right)+\beta_{1}^{*}\Delta t\left(\text{K}⊗\text{I}\right)\left(x,y\right), \end{array}$$

so that the calculation of the second term is reduced to the 2D convolution of the infectiousness matrix **I** with the short term pdf **K**. The calculation of Equation (8) is computationally very demanding, as it has to be done for every susceptible vole for every time step. Fortunately, when we substitute Equation (10) into Equation (8), we can commute the integral to arrive at the following set of equations:

(11) $$\begin{array}{l} \;{\text{P}}_{S\to I}\left(x_{i},y_{i};t\right)=\beta \Delta t{\text{G}}^{*}\left(x_{i},y_{i};t\right),\\ {\text{G}}^{*}\left(x,y;t+\Delta t\right)={\text{G}}^{*}\left(x,y;t\right)\left(1-\xi \Delta t\right)+\Delta t\left({\text{K}}^{*}⊗\text{I}\right)\left(x,y\right),\\ \;{\text{K}}^{*}\left(x,y\right)=\left(\text{K}⊗\text{K}\right)\left(x,y\right), \end{array}$$

where K^*^(*x, y*) is the 2D convolution of two short term Gaussian pdf's **K**(*x, y*). This kernel is again Gaussian, but now with twice the short-term variance (2σ^2^). Hence, including the movement of the susceptible vole itself does not add an extra computational burden, since we simply have to convolve the infectiousness matrix **I** with another kernel, K^*^. On the contrary: since the width of the kernel is slightly larger (a factor $\sqrt{2}$), we can allow the grid cell size to be slightly larger, which reduces the computational burden. Note that the two unknown parameters $\beta_{1}^{*}$ and β_2_ can now be merged into one single unknown parameter β. This fitting parameter will be adapted to fit the simulation results to the field data, as is discussed below. The grid element size Δ*x* = Δ*y* = 7 m is chosen such that K^*^ is sampled sufficiently.

Using this model on a standard pc, we are able to model patches as large as 10 × 10 km, keeping track of and modeling the interaction of more than a million voles.

#### Direct vs. Indirect Transmission

In the above equations, we have assumed that indirect transmission,via virus accumulated in the environment is the most dominant route. As mentioned before, it is also possible that voles get infected through direct contact, e.g., by mating, biting, exploratory sniffing, etc. If one wants to study only direct transmission, one should use:

(12) $$\begin{array}{l} {\text{P}}_{S\to I}\left(x_{i},y_{i};t\right)=\beta \Delta t\left({\text{K}}^{*}⊗\text{I}\right)\left(x,y\right), \end{array}$$

instead of Equation (11).

### Comparing Model Simulations to Field Data

In the following we show simulation results for different model scenarios. For every model scenario, there is only one unknown parameter left which cannot be estimated from the literature: the parameter β, which is related to the efficiency of virus transmission from an infectious bank vole to the ground and from the ground to a susceptible bank vole. In our analyses, we chose to fit β for every model scenario such that the mean peak density of infectious voles in an increase year matched that measured in the field (with resolution of 0.1 voles/ha). The mean peak density of infectious voles in the field was obtained by averaging the two peak densities of infectious voles in the increase years 2004 and 2007 (see Figure 1), and was calculated to be 8.4 bank voles/ha. The model-specific β value was obtained using a simple directed search strategy, in which we iteratively narrowed down to the β that resulted in a mean peak density of infectious voles of 8.4 ± 0.1 bank voles/ha (always marked with a “+” in the figures). This approach allowed us to assess to what extent each model, tuned to produce the mean peak density of infectious voles observed in the field in an increase year, was also able to reproduce the infection dynamics observed in the field during the subsequent peak year.

In order to compare the simulated infection dynamics with the field data and to assess the “quality” of the model, we focused on a selected set of features of the infection dynamics. Our primary feature was the mean peak density of infectious bank voles in the peak abundance year following an increase year. From Figure 1 it is clear that the mean peak density observed in the field (8.8 bank voles/ha; winter 2005 and 2008) was only slightly higher than in an increase year (8.4 bank voles/ha). So the first quality measure was the difference between the simulated peak density of infectious voles in an a peak year and that found in the field (8.8 bank voles/ha); this should be small. Next, we inspected the peak virus accumulation in the environment in a peak year compared to in an increase year. Virus abundance is likely to be related to the number of human NE cases, and Kallio et al. (2009) observed that the number of NE cases in a peak year was similar to or slightly lower than in an increase year. Hence, the second measure is the ratio of the maximal virus accumulation in a peak year to that in an increase year; this should be close to 1. Finally, we quantified the timing of the virus accumulation peaks with respect to the timing of the peak in bank vole abundance. Kallio et al. (2009) observed that the number of NE cases peaked 1–3 months after the peak in bank vole abundance.

In the analysis, we focus on these three features and discuss to what extent these are in agreement with the field data (using the quality measures). We deliberately refrained from an elaborate statistical evaluation of the quality of the different models, because we have only two full 3-years cycles of field data to compare the model output with, and consequently any statistical test would lack power. One could argue that one cycle consists of multiple data points, but these data points are highly correlated in time. Moreover, the simulated bank vole density shown in Figure 2C is only an approximation of the abundance measured in the field, and the approximation is worse during periods of low density between peaks (remember the carrying capacity was chosen to match the peak values), which makes a detailed analysis of the data throughout the year not meaningful.

## Results

All simulations were done on an *in silico* 3 × 3 km grid for 105 3-years cycles (315 years), of which only the last 100 were used for the analyses, as the simulations are then no longer affected by the artificial initial starting conditions. To indicate the stochastic nature of the simulations, the results are shown as “bands” centered around the averages over these 100 cycles, with a width that is twice the standard deviation. Hence, the broader the band, the higher the stochastic variation at this point in the cycle. The simulation results throughout the 3-years cycles are shown as separate figures for every scenario, and the “+” always marks the same infectious bank vole density level (8.4 voles/ha) that was used to fit all models (through β, see Table 1). The virus quantity was scaled such that the mean virus quantity in an increase year equals 1, since we are only interested in the relative height of the peaks. The extracted features (peak value of the density of infectious voles, peak value of the (scaled) virus accumulated in the environment and the timings of the virus peaks) are listed in Table 1.

**Table 1:** The first column shows the different values for the model parameter β such that the maximal density of infectious bank voles in the increase year equals 8.4 bank voles/ha (marked by “+” in Figures 5, 6) for the different models.

**Model**	**β(year^−1^vole^−1^)**	**Maximal density of infectious voles in peak year**	**Maximal viral load in peak year**	**Time delay in increase year (months)**	**Time delay in peak year (months)**
Figures 5A,B	16,400	16.6	1.66	1.9	1.0
Figures 5C,D	16,400	16.2	1.62	1.9	1.0
Figures 5E,F	59,000	11.3	0.99	2.0	1.4
Figures 5G,H	59,000	11.0	0.96	2.0	1.5
Figures 6A,B	59,000	11.0	0.96	2.0	1.5
Figures 6C,D	300,000	10.2	1.33	1.2	1.0
Figures 6E,F	29,000	10.1	0.64	3.4	3.7

*The data of the other columns correspond to the different features of the simulated infection dynamics that are considered in the analyses. These values are compared with their respective values measured in the field: maximal density of infectious voles in a peak year: 8.8 voles/ha; maximal viral quantity (number of NE-cases) in a peak year: in general <1; the delay between bank vole abundance peaks and virus quantity peaks (number of NE-cases): between 1 and 3 months*.

### No Immunity, No Density-Dependent Spatial Behavior Between Years

**Figure 5:**
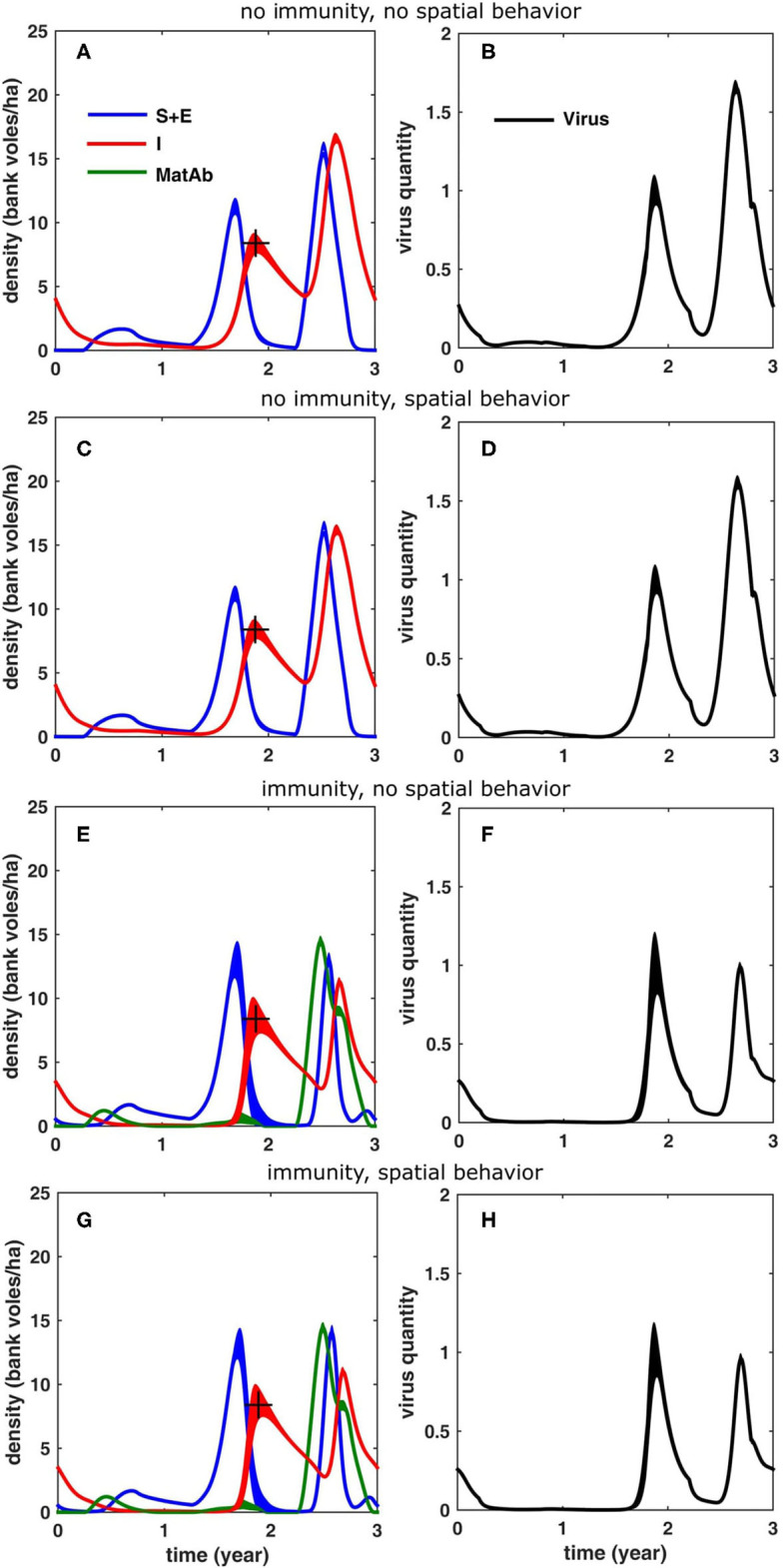
Simulation results for a 3-years cycle (averaged over 100 cycles; the width equals twice the standard deviation) for the different scenarios, see text: **(A,B)** without reduced home range in the peak year and without inclusion of immunity due to maternal antibodies, **(C,D)** with reduced homerange only, **(E,F)** with inclusion of immunity only and **(G,H)** with inclusion of both immunity and reduced homerange. The figures in the left column **(A,C,E,G)** show the density of bank voles with the respective infection statuses (blue: susceptible and exposed, red: infectious, green: protected by MatAb); the figures on the right **(B,D,F,H)** show the corresponding amount of virus that was accumulated in the environment. The “+” marks the infectious bank vole density level (8.4 voles/ha) that was used to fit the model (through β, see Table 1).

First, we ran simulations without including immunity or density-dependent spatial behavior. The immunity period was set to zero, such that antibodies were cleared after the first day (i.e., effectively removing the effect of maternal antibodies). The spatial behavior only varied within the year (σ = 10 m for all voles, except during the breeding season, when σ for males and females temporarily increased to σ = 16 and 32 m, respectively) but did not differ between years. We assumed indirect virus transmission (via the environment) and an acute and a chronic infectious phase where virus shedding of infected individuals is lower (10%) in the latter. The simulation results are shown in Figures 5A,B. Comparing the results to those measured in the field, it is clear that the simulated density of infectious voles reached much higher values in a peak year (16.6 bank voles/ha) compared to the increase year (≈2 times as high) (see also Table 1). Also, the corresponding accumulation of virus was much higher (1.66 times) in the peak year, compared to the increase year (see Figure 5B). The accumulated virus in the environment attains its maximum with a delay of about 2 months in an increase year and 1 month in a peak year (following to the peak vole density).

### Including Density-Dependent Spatial Behavior

Next, we considered the effect of inter-annual variation in density-dependent spatial behavior of bank voles on the infection dynamics. Again, we assumed that there is no maternally-derived immunity, but now, in a peak year, we reduced the home ranges of female and male voles, respectively to σ = 10 and 16 m during the breeding season. The results in Figures 5C,D show that the temporary decrease of home range size during a peak year had only a minor effect. It resulted in a slight reduction of the peak density of infectious voles (16.2 bank voles/ha) and of the peak environmental virus contamination (1.62); the effect on the timing of the peaks was also minor; the simulation results were very similar to those shown in Figures 5A,B.

### Including MatAb Immunity

To study the effect of immunity on the infection dynamics, we assumed that the voles are protected for about 80 days, as found by Kallio et al. (2006b). In order to compensate for the hampering effect of the immune bank voles the model required a higher β−value (see Table 1). As is clear from Figures 5E,F, including immunity had a profound effect on the infection dynamics, especially in a peak year, when the influx of immune voles suppressed the peak density of infectious voles (11.3 bank voles/ha). A similar effect was observed for the environmental virus contamination, which was slightly lower than in the increase year (0.99). The peaks were also attained slightly later in the peak year (1.4 months). The model outcome was not sensitive to the exact duration of the immunity period, except for the density of immune voles.

If, in addition to immunity, we also include the density-dependent spatial behavior, the results in Figures 5G,H show that the temporary reduction in home range size further reduced the density of infectious voles (11 bank voles/ha), and, similarly, further suppressed the maximum environmental contamination during the peak year (0.96), but the overall effect was small. The timing of the peaks were also only slightly affected.

### If Immune Animals Are Under-Diagnosed

Comparing the simulation results to the field data shown in Figure 1, it is clear that for the scenarios including immunity (Figures 5E–H), the density of immune voles was larger than estimated from field data. In order to understand why such low levels of immune animals were found in the field, we took a closer look at the way these immune animals are identified. In their longitudinal study in which they followed offspring from infected mothers, Kallio et al. (2006b) found that not a single (initially protected) vole got infected before day 80. Apparently, maternal antibodies had sufficiently high levels to provide protection. It was only at the next testing event, on day 105, that many of these formerly protected animals were infected. Infection was identified through seroconversion events, i.e., if a blood sample was positive and the previous blood sample had been negative. But the authors also found that: “maternal antibodies disappeared gradually by the age of 80 days, when none of the MatAb positive animals had detectable antibody levels.” Since we know that they were still protected at this age, it seems that a vole can still be protected even when antibody levels providing this protection are below the detection threshold of an immuno-fluorescent assay (IFA). As a result, some voles that are still protected by antibodies but have attained low MatAb levels will be misclassified as being susceptible, i.e., free of antibodies.

**Figure 6:**
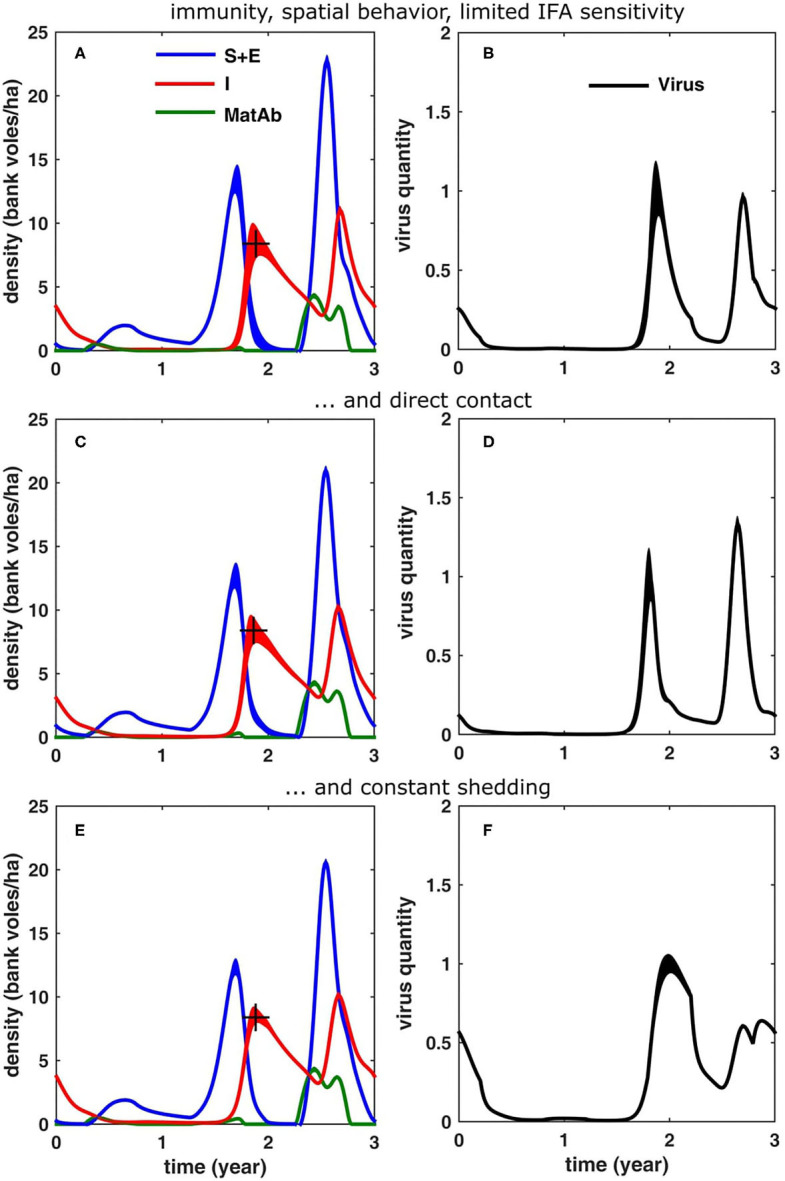
Simulation results (averaged over 100 cycles; the width equals twice the standard deviation) for different scenarios, see text: **(A,B)** same as in Figures 5G,H, but now if it is assumed that the immuno-fluorescent assay (IFA) has a limited sensitivity to maternal antibodies (MatAb); **(C,D)** if only direct transmission is assumed and **(E,F)** if the virus shedding does not decrease over time.

In order to test what the effect of this would be, we have plotted in Figures 6A,B the results for the case that antibody levels can be detected only until 2 weeks after birth. These show that the simulation results (regarding the density of susceptible and immune bank voles) were now in better agreement with the densities of the respective infection statuses, as they were measured in the field.

### Direct vs. Indirect Transmission

In the above simulations, we have considered only the indirect route (Kallio et al., 2006a), as this is thought to be most important one (an assumption which may be biased by the fact that humans only get infected through this route). In order to investigate how direct transmission (e.g., through biting, sniffing, mating, etc.) would alter the infection dynamics, we have run the same simulations as in Figures 6A,B, but now assuming infection through direct contact instead of indirect contact. Assuming direct contact reduced the mean density of infectious voles (10.2 bank voles/ha). It resulted in a peak contamination which is higher in the peak year compared to the increase year (1.33) and “sharpened” the peaks of accumulated virus. Apparently, the force of infection is larger in the case of indirect transmission and the infection spreads more rapidly, reducing the time delay in an increase year to 1.2 months.

### Decreasing vs. Constant Shedding

In all previous simulations, we have assumed that the voles go through an acute phase of about a month, followed by a chronic infectious stage which lasts for the rest of their life. In case the amount of virus shed does not decrease over time but remains constant, the results are shown in Figures 6E,F. The infection dynamics were again qualitatively similar to Figures 6A,B, although assuming direct contact reduced the mean peak density in the peak year (10.1 bank voles/ha). The virus build-up in the environment showed a different pattern, though, especially in the peak year: the maximal contamination levels were much lower (0.64) and both in the increase and peak year, the virus contamination peak was attained later in the year (delay of 3.4 and 3.7 months, respectively), i.e., with an additional delay of ≈1.5 months. Also, in general the peaks were much broader.

## Discussion

Incorporating the effect of maternal antibodies on zoonotic infection dynamics has long been neglected (Boulinier and Staszewski, 2008), but its relevance has now been described for several zoonotic disease systems besides hantaviruses (Plowright et al., 2008; George et al., 2011). The simulation results confirmed that it is necessary to include maternal passive immunity to explain the infection dynamics of hantavirus in Central-Finland. Chronic infection patterns only, as suggested by Sauvage et al. (2003), were not sufficient to explain the observed transmission dynamics in this study region: they resulted in much higher abundances of infectious voles in a peak year compared to an increase year, contrary to what was found by Kallio et al. (2010). Including maternally-derived immunity in the dynamics reduced the density of infectious bank voles in the peak year to levels similar to those in the increase year (Kallio et al., 2010). It was found that the smaller home ranges during a peak year could not account for the reduced number of infectious bank voles in peak abundance years; the effects of density-dependent variation in home range size were minor. However, it should be noted that assessing the impact of density-dependent home range sizes requires a model with higher complexity, compared to the mere inclusion of transient immunity, and consequently asks for non-trivial assumptions (e.g., on how the bank vole moves within its home range, the shape of the home ranges, etc.) Having such information available could further strengthen the inference of our conclusions.

Our model simulations also help explaining the occurrence of NE in the human population in Central-Finland during the same time period. In line with the pattern observed for the abundance of infectious voles, Kallio et al. (2009) found that “the obvious difference in bank vole abundances between increase and high years is not reflected in the human NE epidemics. In fact, human peaks were slightly higher in the increase (2001 and 2004) than in the high (2002 and 2005) phase of vole cycles.”According to our simulations, this can mainly be attributed to an immune juvenile dilution effect at the start of the breeding season, reducing the magnitude of virus contamination (and hence the risk for humans) in a peak vole year to a level similar to that in the increase year. Moreover, the peak in virus contamination follows the peak vole abundance with a delay of about 1–2 months (except in case of constant shedding), which is also in agreement with the human NE incidence pattern described by Kallio et al. (2009), who observed a delay of 1–3 months.

Although the density of infectious voles in a peak year matched that measured in the field, the simulated density of immune voles was much higher than measured in the field. This difference may be due to the limited sensitivity of the antibody assay to detect MatAb immune voles (Gilbert et al., 2013). Although animals are protected for around 80 days, antibodies may only be detected for a much shorter period which would reduce the number of immune voles detected. Moreover, it is likely that mainly very young animals will have detectable antibody levels and those animals are less likely to be captured in the field because they stay in the burrows, and this would result in an important underestimation of the immune young animals. But, this of course is a conjecture. A study of the sensitivity of the MatAb test could further substantiate (or refute) the validity our model outcome.

Bank voles can transmit an infection through direct or indirect contact (Kallio et al., 2006b), but it is not yet clear which route is responsible for most of the infections. Although the indirect route seems to be most likely and was therefore included in most of the simulations, assuming direct transmission in the model resulted in similar infection dynamics. From a modeler's perspective, this means that the model output is not that contingent on which route is dominant, since both models produce similar results. Note however that in this model we have focused on the dynamics in the increase and peak years, and not on persistence in low abundance years. In the case of low host abundances, it may be that one of the routes is essential for the infection to persist through longer periods of low abundance. The conclusions are slightly different when it comes to the amount of virus accumulated in the environment. It was found that assuming direct transmission favors virus accumulation in a peak year, increasing it to a level slightly above that in the increase year, which is somewhat contrary to what the NE-incidence data suggest.

Related to this, there is still no conclusive evidence on the exact shedding pattern that has to be taken into account (Hardestam et al., 2008; Voutilainen et al., 2015). The patterns are different for blood, feces, urine and saliva, and moreover, we do not know which decrease of virus shedding would be biologically relevant. Therefore, to test the sensitivity of the model output to the exact shape of the shedding pattern, we also ran model simulations assuming constant shedding. This model produced similar infection dynamics of the bank voles, regarding the peak densities of infectious voles in the peak year. The major difference was in the accumulation of virus in the environment: the amount of accumulated virus was much lower in a peak year, and peak values were attained more than 3 months after the peak abundance, which is somewhat later than observed in the NE-incidence data.

It is plausible that in reality both indirect and direct routes are to some extent used for virus transmission and the “effective” pattern of virus shedding is somewhere in between the two considered shedding patterns. In fact, there are likely seasonal differences in the role of direct (breeding in summer) and indirect (non-breeding in winter) transmission and since the temporal change in virus presence is different in blood, feces, urine and saliva, it is to be expected that the shedding patterns too may be different for both routes.

The model used here improved on previous models by allowing complex spatial dynamics of the bank vole/virus interaction: it models the spatial behavior (dispersal and varying home ranges) of the bank voles explicitly, in an environment that is being contaminated with (and gradually cleared from) virus. Apart from the particular situation in Central-Finland, the model can be used for studying the distribution of PUUV throughout Europe. The model, attuned to (the best documented situation in) Central-Finland, can be used as a baseline to investigate to what extent the variation of different system (model) parameters alters the infection dynamics, and to identify processes responsible for the heterogeneous distribution of PUUV across Europe. Finally, the applicability of the model is not limited to PUUV, as it could easily be adapted to model the spatial transmission dynamics of other pathogens. It is especially suitable to study infections in which the variable spatial dynamics of the host are thought to play an important role in the transmission dynamics.

## Conclusion

In this paper, we have advanced a novel spatially-explicit model to simulate PUUV infection dynamics in a population of bank voles. We have applied the model to the specific situation in Central-Finland, where PUUV infection occurrence is generally high and poses a significant threat to humans (Vaheri et al., 2013). Using the model, we addressed the question of why the abundance of infected bank voles in Central-Finland bank voles does not necessarily increase with host abundance, i.e., why the PUUV infection level in a peak year is similar to that in the preceding increase year. The simulation results confirmed that it is necessary to include maternal passive immunity in the infection dynamics, to reduce the density of infectious bank voles in the peak year to levels similar to those in the increase year. The simulation results produced larger numbers of immune animals than generally found in the field, though, which may be attributed to a lack of sensitivity of the antibody assay and a trapping bias toward older bank voles, who are more likely to have lost their immunity. Maternally-derived immunity in bank voles can also explain the patterns of occurrence of NE in the human population, both with respect to the relative numbers of NE cases, as to the timing of the peaks. The effect of density-dependent variation in home range size was shown to be very limited and could not account for the infection dynamic patterns observed. Finally, it was shown that including indirect transmission in the infection dynamics did not change the above conclusions, neither did assuming a constant shedding pattern. Hence, the conclusions drawn from these model simulations are robust and not too sensitive to the exact system parameters.

## Data Availability Statement

The datasets analyzed in this article are not publicly available. Requests to access the datasets should be directed to heikki.henttonen@luke.fi.

## Author Contributions

JR, KT, BB, and NH designed the model, based on input on bank vole and Puumala virus ecology provided by LV, HH, and HL. JR implemented the model, performed the simulations, and wrote the first draft of the manuscript. LV and HH provided field data. All authors contributed to, read, and approved the submitted version.

## Conflict of Interest

The authors declare that the research was conducted in the absence of any commercial or financial relationships that could be construed as a potential conflict of interest.
