# Supplementary material for: Why Hantavirus Prevalence Does Not Always Increase With Host Density: Modeling the Role of Host Spatial Behavior and Maternal Antibodies
Source: Front Cell Infect Microbiol. 2020 Sep 29;10:536660. doi: 10.3389/fcimb.2020.536660 (PMC7550670; doi:10.3389/fcimb.2020.536660)
Supplement: Supplementary file 2 [file Data_Sheet_2.PDF]

Supplementary information

Jonas Reijniers, Katrien Tersago, Benny Borremans,
Nienke Hartemink, Liina Voutilainen, Heikki Henttonen, Herwig Leirs

1 Prevalence

In Fig. S1 we have plotted the prevalence and the bankvole density, corresponding to Fig. 1. Note that the model could also have been fitted based on prevalence, instead of density of infected animals. However because prevalence is highly dependent on total population density, it is much more sensitive to minor changes in population density, in particular during periods of low density. We therefore chose to base our models on the density of infected animals.

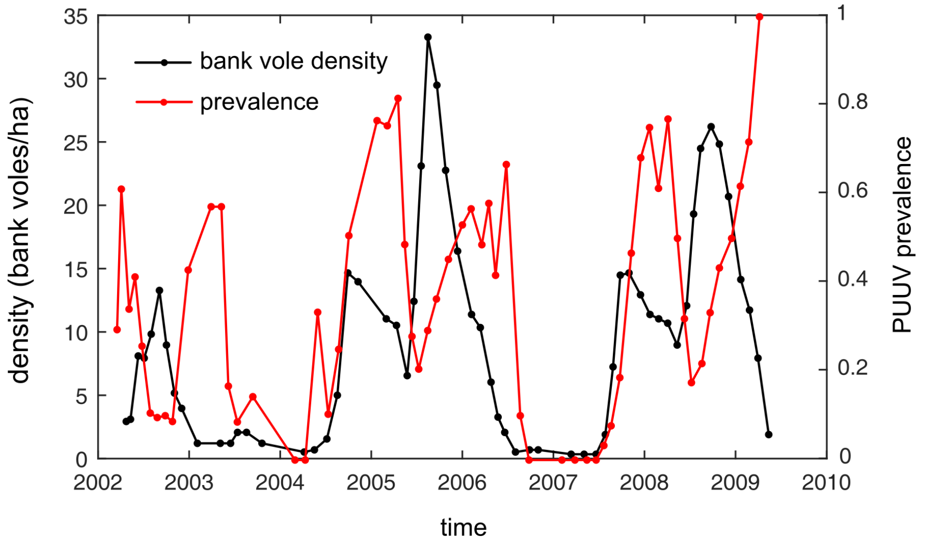

Figure S1: prevalence vs the bank vole density, corresponding with the field data shown Fig. 1.

2 On the shedding pattern

In previous models, it was assumed that infected bank voles never fully recover from PUUV infection and remain chronically infected. Following the shedding data collected by Hardestam et al. [1], two different infectious stages where assumed: an acute phase of about a month, and a chronic phase, in which lower amounts of virus were excreted. The data were collected in a laboratory setting. In a recent manuscript, Voutilainen et al. [2] presented data on the temporal dynamics of virus concentration in saliva, faeces and urine for bank voles capured (and recaptured) in the field. These data are shown in

Fig. S2(a,c,e). In this analysis, we consider virus concentration on a linear scale (instead of the logarithmic scale that was previously used), since we assume that the probability for being infected is proportional to the amount of virus particles that a bank vole is confronted with.

The problem with the data on a linear scale is that the residuals of the data are not normally distributed and consequently one cannot make use of parametric statistical tests. Given the limited number of datapoints available, this limits the complexity of the statistical models and the hypotheses that can be tested. Therefore, in the following, we only consider the rather simple hypothesis that the virus shedding in the acute phase (< 30 days after seconversion) is larger than that in the chronic phase (> 30 days). To this end, we make use of the permutation test, a robust nonparametric test which does not impose any requirements on the distribution of the residuals. We discuss the permutation test in case of saliva, illustrated in Fig. S2(a) and (b); a similar test was done for faeces and urine.

For saliva, we have 100 individual datapoints, of which 44 were measured before and 56 after day 30. We calculate the respective means, and take the difference. We find that

$$\begin{aligned} \text{mean(acute phase)} &= 2339 \\ \text{mean(chronic phase)} &= 263, \end{aligned}$$

hence the difference in means is 2076.

Now, with the permutation test, we want to assess the probability that this difference is due to sheer chance. To this end, we permute the datapoints (and consequently, neglect the time since seroconversion), and randomly split the dataset in sets of 44/56 points, we calculate the respective means and take the difference. And this we do for a large number of times (100000). Next, we can make a histogram of the difference in mean, in case the time since seroconversion is unimportant, shown in Fig. S2(b). When locating the actual difference of means that was actually measured (2076), shown by the red dot, we find it to be situated in the tail of the distribution. Calculating the cumulative function, we find that the probability that, if the null hypothesis is true and there is no difference in shedding between acute and chronic shedding, the probability to arrive at the measured difference of means (or larger) is $p = 1 - 0,9936 = 0,0064$. Hence, from this we can conclude that the concentration in saliva before and after day 30 are significantly different ($p < 0.05$).

A similar permutation test was done for the other excrements, urine and faeces, and is shown in Fig. S2(c,d) and (e,f) respectively. Here, the difference in shedding patterns in the acute and chronic phase could not be proven to be significant. The reason for this could be that the shedding dynamics are not different and the bank voles continue to shed similar amounts of virus after they have been infected. But another reason may be the lack of statistical power of the dataset. Because the concentrations of virus in faeces and urine are much lower compared to in saliva, which make the data more prone to noise. But also because the number of datapoints is smaller, especially in case of urine (only 39 datapoints).

Because of the ambiguous nature of the results, in the manuscript we considered two situations, (1) where we distinguish acute from chronic shedding, where latter falls back to approximately 10% of the original shedding and (2) where the shedding remains constant after infection.

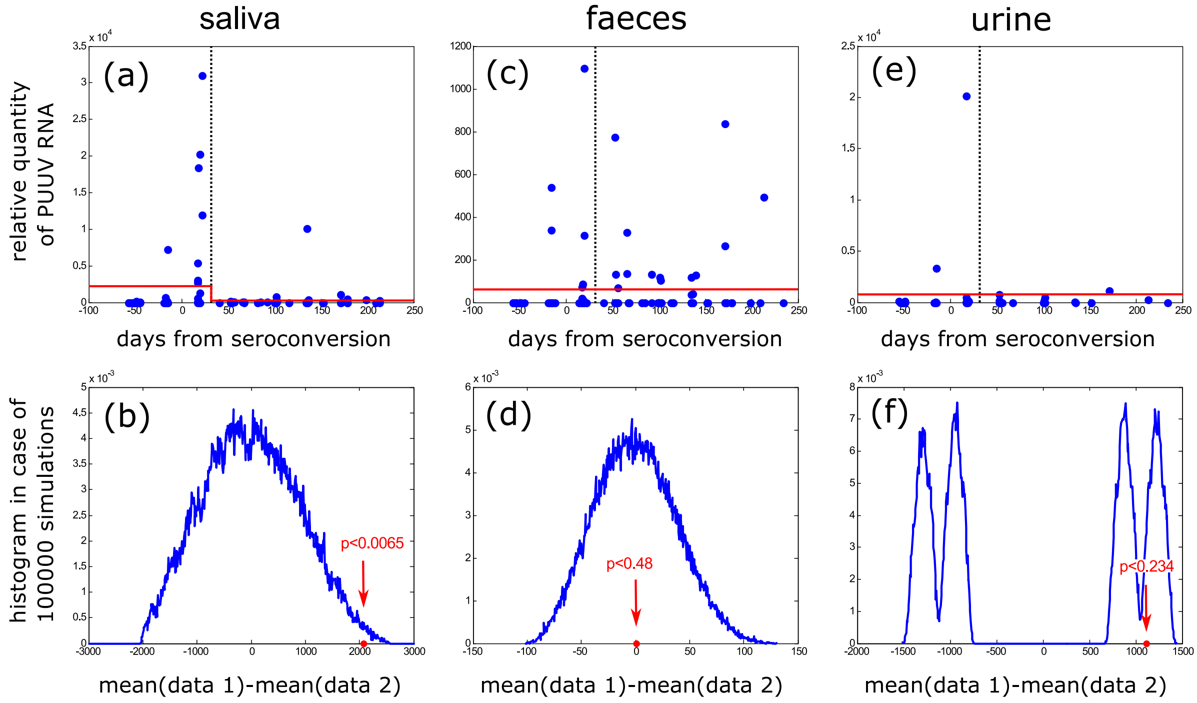

Figure S2: The relative quantity of PUUV RNA as function of the estimated date of seroconversion, in (a) saliva, (c) faeces and (e) urine, see Ref. 2. The dashed lines separate the hypothesized acute from the chronic phase (at 1 month after seroconversion). The red lines indicate the mean relative quantity in the acute and chronic phase in case the difference is significant (a), if this is not the case (c and d) the overall mean is shown. In (b), (d) and (f) the respective distributions of the difference of means of 100000 permutations is shown. The red dot corresponds to the difference of means in the nonpermuted dataset. The corresponding p-value is also indicated in red.

3 Mean field approximation

From the simulation results shown in Fig. 5(c,d) and (g,h) it is clear that the effect of a reduced homerange in the peak year has only a small effect on the infection dynamics. Hence, the question arises if it was necessary to model the spatial dynamics into such great detail, or a mean field approximation would have sufficed. To test this, we have ran the simulations for the model in Fig. 5(g,h), i.e. with maternal immunity and reduced homerange, but now we distributed the virus quantity evenly over the grid, by taking the average over the grid. This way, the spatial dynamics is no longer included in the infection dynamics. (Note that we did not remove the spatial dynamics when modelling the population dynamics, otherwise we would arrive at a different population density curve).

The results are shown in Fig. S3 and Table S1. Assuming random mixing produces clearly different results than shown in Fig. 5(g,h). First, it requires a smaller β, as is to be expected since the susceptible bank voles are no longer depleted locally. Second, he peak density of infectious voles in the peak year is reduced, and so is the corresponding virus quantity peak. Last, the virus quantity peaks are delayed with an additional 0.5 months.

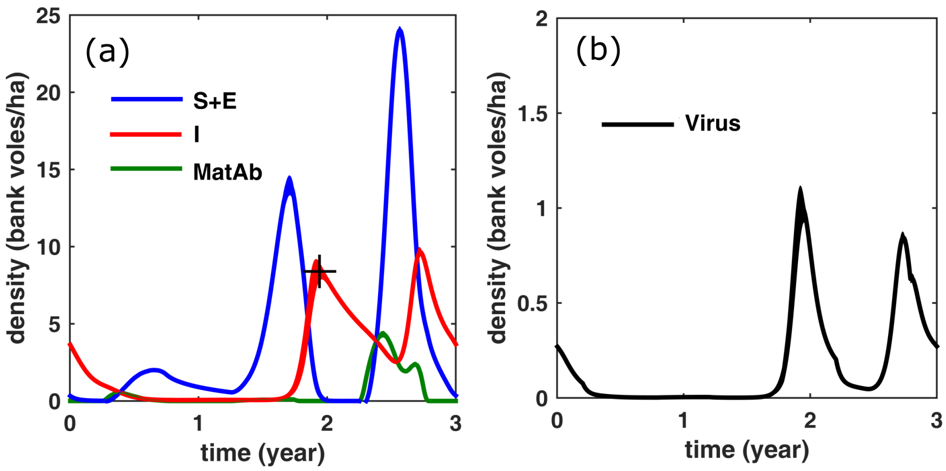

Figure S3: Same as in Fig. 5(g,h), but now in the mean field approximation, i.e. if the virus load is distributed evenly over the area.

model	β (year^{-1}vole^{-1})	maximal density of infecteds in peak year	maximal viral load in peak year	time delay in increase year (months)	time delay in peak year (months)
Fig. S2(a,b)	31000	9.7	0.84	2.7	2.0

Table S1: The different values for the model parameter β such that the maximal density of infectious bank voles in the increase year equals 8.4 bank voles/ha, for the random mixing model. The data of the other columns correspond to the different features of the simulated infection dynamics curves which are considered in the analyses.

References

[1] Hardestam J, Karlsson M, Falk KI, Olsson G, Klingström J, Lundkvist A (2008) Puumala hantavirus excretion kinetics in bank voles (Myodes glaterolus), Emerg Infect Dis 14 (8): 1209-1215.

[2] Voutilainen L, Sironen T., Tonteri E, Tuiskunen Bäck A, Razzauti M, Karlsson M, Wahlström M, Niemimaa J, Henttonen H, Lundkvist A (2015) Life-Long Shedding of Puumala Hantavirus in Wild Bank Voles (Myodes glareolus). J Gen Virol 96, 1238–1247.
